# Impacts of Type D Personality and Depression, Alone and in Combination, on Medication Non-Adherence Following Percutaneous Coronary Intervention

**DOI:** 10.3390/ijerph15102226

**Published:** 2018-10-11

**Authors:** Youn-Jung Son, Kyounghoon Lee, Donald E. Morisky, Bo-Hwan Kim

**Affiliations:** 1 Red Cross College of Nursing, Chung-Ang University, Seoul 06974, Korea; yjson@cau.ac.kr; 2 College of Medicine, Division of Cardiology, Gachon University, Incheon 21565, Korea; cardioman@gilhospital.com; 3 Department of Community Health Sciences UCLA Fielding School of Public Health, Los Angeles, CA 90095-1771, USA; dmorisky@ucla.edu; 4 College of Nursing, Gachon University, Incheon 21936, Korea

**Keywords:** Type D personality, depression, medication non-adherence, percutaneous coronary intervention

## Abstract

*Background*: Medication adherence after percutaneous coronary intervention (PCI) is essential to preventing the risk of restenosis. Even though Type D personality and depression have been known to affect medication non-adherence, their combined influence on PCI patients remains unclear. *Aim*: We aimed to identify how both Type D personality and depression were associated with medication non-adherence for 3 months after successful PCI. *Methods*: This prospective cohort study included 257 PCI patients, who took 3 or more cardiac medications, at a university hospital. We measured sociodemographic and clinical variables, Type D personality, depression, and medication non-adherence using face-to-face interviews and medical record reviews. *Results*: The total prevalence of medication non-adherence at the one- and three-month follow-ups was 14% and 16%, respectively. At one month, the prevalence of those with a combination of Type D personality and depression (23.4%) and depression alone (24%) was significantly higher than other groups. At three months, the prevalence of the Type D personality-only group (39.1%) was the highest. Type D personality increased the risk of medication non-adherence 5.089 times at three months, while depression increased it 2.6 times at one month. However, the risk of medication non-adherence was not increased in patients with combined Type D personality and depression. *Conclusions*: Individual assessments of Type D personality and depression are required. Therefore, psychological interventions focusing on personality and depression are crucial. Longitudinal follow-up studies must explore the interaction or individual impact of Type D personality and depression on medication non-adherence and other negative outcomes.

## 1. Introduction

Percutaneous coronary intervention (PCI), using a balloon and stent in the diseased area of the artery, is effective in relieving symptoms such as chest pain, shortness of breath, and nausea [1]. By treating the stenotic coronary arteries, it also prevents the risk of new cardiac events and early death in patients with cardiovascular disease [1]. Discharge from hospital is a critical time for patients because they need to adjust their lifestyles and incorporate new medications [2].

Medication adherence is a particularly important component of self-care after PCI for effective secondary prevention [3]. In addition, medication adherence significantly improves health outcomes, and has even been found to reduce total annual coronary artery disease (CAD) medical costs [2,4]. Approximately 50% of patients with cardiovascular disease display poor adherence to prescribed medications [5,6]. According to a previous study, the percentage of patients adherent to both antihypertensive and lipid-lowering therapy declined sharply following treatment initiation, with 44.7%, 35.9%, and 35.8% of patients adherent at 3, 6, and 12 months, respectively [7]. According to another study, patients who were not compliant with four or more cardio-related medications had about a three times higher risk of mortality and reinfarction rates [8].

Non-adherence to medications remains a major problem for cardiovascular patients. It leads to poor clinical outcomes, including rehospitalization, subsequent myocardial infarction (MI), and increased mortality in various patient settings [9]. To improve patients’ short- and long-term medication adherence after PCI, healthcare providers need to use multiple approaches [10].

Type D personality, a type personality, is combined by social inhibition (SI, the tendency to inhibit the expression of these emotions in social interaction) and negative affectivity (NA, the tendency to experience the negative emotions) [11]. The Type D personality concept was originally developed to identify cardiac patients at risk of developing emotional and interpersonal difficulties [12]. It has been strongly associated with risk factors of poor perceived health patients in cardiac disease [11,12]. For this reason, Type D personality has been included in the European Cardiovascular Prevention guideline since 2012 [13]. The prevalence of Type D personality among CAD is around 20–50%, making it a factor that should not be ignored [14]. Type D personality has been strongly linked to both physical and psychological morbidity and survival after the onset of conditions related to cardiovascular disease [15]. Those with Type D personality showed an increased risk of major adverse cardiac events such as MI, PCI, and coronary artery bypass graft at 15 months post-PCI [16].

The prevalence of depression as a mood status in patients with CAD ranges from 25–50% [17]. Depression after a coronary artery bypass graft was a predictor of higher readmission rates within 6 months and increased cardiac events and mortality [18]. Depression not only reflects episodic distress but also a more ingrained tendency to experience distress, with the combination of distress and social isolation predicting a poor cardiac prognosis [19].

Despite evidence of these factors’ importance in the outcomes of CAD patients, the prospective relationship between medication non-adherence and Type D personality or/and depression has rarely been reported [20]. Moreover, there have been debates regarding overlapping constructs between Type D personality and depression [21,22]. One study reported that the negative affectivity dimension, measured through the Type D Scale-14 (DS-14), and depressive symptoms are largely overlapping constructs [22]. Conversely, most previous studies [23,24,25,26] have confirmed that Type D personality and depression represent a different form of distress. Namely, Type D personality traits have been found to be relatively stable over time and their combination gives rise to a general propensity to psychological distress such as depression [23,24,25,26]. Throughout previous studies, if Type D personality might affect depression, and if Type D personality and depression might be present together, it is likely to have a greater negative effect on medication adherence.

To date, even though CAD has become a long term chronic condition, little is known for combined Type D personality and depression in patients after PCI [20,27]. Therefore, to improve adherence to medication after PCI, it is essential to investigate longitudinally the association of Type D personality and depression with medication adherence. Therefore, this study aimed to identify the rates of adherence to prescribed medication and then to explore the relationships between the medication non-adherence and combined Type D personality and depression after successful PCI.

## 2. Methods

### 2.1. Study Design and Setting

This was a secondary analysis of data from a prospective cohort study demonstrating that left ventricular ejection fraction and post-cardiac depressive symptoms have a combined effect on major adverse cardiac events after successful primary PCI during a 12-month follow-up in Korea [28]. We used G-power program 3.1 version to determine a sufficient sample size using an alpha of 0.05, a power of 0.95, a medium effect size (odd ratio = 1.72) and two-tailed test. Based on the aforementioned assumptions, the desired sample size was 286. For 3 months, 257 patients of primary study [28] completed the follow-up. In brief, participants in this prospective study were patients with CAD at Soonchunhyang University Hospital of Cheonan in Chunggnam, South Korea between July 2011 and January 2012. Data were collected from July 2011 to February 2013. We chose 3 months as the minimum follow-up period for two reasons. First, medication adherence had significantly decreased at 3 months, to the point of affecting mortality or readmission [7,27]. Second, patients who had undergone PCI had been diagnosed with Type D personality or depression, which might have affected medication adherence [18,20].

Inclusion criteria included being on three or more cardiac medications (including any of the following: antiplatelets, *β*-blockers, angiotensin-converting enzyme inhibitors, angiotensin II receptor blockers, nitrates, calcium channel blockers, antiarrhythmics, diuretics), aged 18 years or above, fluent in Korean, and able to provide informed consent. Patients were excluded from the study if they had cognitive impairment, known alcohol or illicit drug use, or a physical or psychological disability inhibiting communication. At baseline, patients completed measures of Type D personality and depression and provided demographic characteristics. At 1 and 3 months, patients completed a self-reported measure of medication adherence via a face-to-face interview at the outpatient clinic.

### 2.2. Measures

#### 2.2.1. Sociodemographic and Clinical Variables

Based on previous studies, we selected sociodemographic and clinical variables that could cause medication non-adherence [27,29]. The baseline sociodemographic variables considered for this study were age, sex, educational level, marital status, religion, income, job, medical insurance, exercise, smoking, and alcohol consumption. The clinical variables considered were medical diagnosis, type of PCI (balloon angioplasty or stenting), and patient history obtained from medical records and interviews.

#### 2.2.2. Medication Adherence

Medication adherence was measured at the 1- and 3-month follow-up hospital visits using the Morisky Medication Adherence Scale-8 (MMAS-8) developed and translated Korean language version by Morisky, Ang, Krousel-Wood, and Ward [30,31,32]. The MMAS-8 was designed to facilitate identification of barriers to and behaviors associated with adherence to hypertensive medication.

It is a self-report questionnaire with 8 questions (items). Medication non-adherence is detected by an MMAS-8 score <6 and medication adherence is detected by an MMAS-8 score ≥6. Cronbach’s *α* for the MMAS-8 was 0.83. In this study, Cronbach’s *α* was 0.58.

#### 2.2.3. Type D Personality

For Type D personality, the DS-14 developed by Denollet [33] was used. We adopted the validated Korean version of the DS-14 [34]. The Type D Scale measures two stable personality traits, that is negative affectivity (7 items) and social inhibition (7 items). Items are rated on a scale from 0 (false) to 4 (true). Respondents scoring 10 or higher on both subscales are classified as having Type D personality [33]. In this study, Cronbach’s *α* for the scale was 0.95.

#### 2.2.4. Depression

The Patient Health Questionnaire (PHQ-9) is a 9-item self-reported questionnaire designed to evaluate the presence of depressive symptoms over the prior two weeks [35]. In this study, we adopted the Korean version of the PHQ-9 standardized by Choi et al. [36]. Developed by Spitzer, Kroenke, and Williams [37] for the purpose of major depression diagnosis, the items of the PHQ-9 are rated on a scale of 0 to 3, with higher scores reflecting greater depressive symptoms. PHQ-9 scores of 0 to 4, 5 to 9, 10 to 19, and 20 to 27 reflect no, mild, moderate, and severe symptoms, respectively. In this study, we set the cutoff score for the depression group at a PHQ-9 score of 5 or more [36] and Cronbach’s *α* for the scale was 0.84.

### 2.3. Statistical Analysis

All analyses were undertaken using SPSS Version 23 (IBM Corporation, Armonk, NY, USA). *p* values < 0.05 were considered statistically significant. Categorical data were presented as percentages and continuous data as mean (SD). Differences between continuous variables were assessed using a *t*-test, and the *x*^2^ test (or Fisher’s exact test when appropriate) was used for categorical variables.

To measure medication adherence, we used a repeated measures analysis of variance (ANOVA) and a one-way ANOVA performed by combined Type D personality and depression groups. Univariate and multivariate logistic regression analysis was conducted with medication adherence at 1 and 3 months as the dependent variable. We adjusted for age, sex (male = 1, female = 0), educational level (low = 1, high = 0), exercise (no = 1, yes = 0), and perceived health status (low = 1, high = 0).

### 2.4. Ethical Considerations

The investigation conforms to the principles outlined in the Declaration of Helsinki. Ethical approval for secondary analysis was granted by the Institutional Review Board of Gachon University in May 2018 (IRB No.1044396-201804-HR-087-01). Patients were given an information sheet about the study by cardiology staff who would normally be involved in their care. If they wished to participate, a meeting was set up with a researcher where further information about the study was provided and written informed consent was obtained.

## 3. Results

### 3.1. Patients’ Characteristics at Baseline

A total of 257 patients who underwent PCI were included in this study (Table 1). The total prevalence of Type D personality and depression was 19% and 28% in patients with CAD, respectively. The breakdown was as follows: 162 (63%) participants in the non-Type D personality and non-depression group; 23 (9%) in the Type D personality-only group; 47 (18%) in the depression-only group; and 25 (10%) in the Type D personality and depression group. The average age of participants was 62.32 ± 10.49 years. More than half the patients were male. At 82.6%, the proportion of males was the highest in the Type D personality-only group (*p* = 0.044). The lowest educational level was observed in the combined Type D personality and depression group (*p* = 0.001). At 24%, the proportion of patients who exercised (*p* = 0.010) was the lowest in the Type D personality and depression group.

Among clinical variables, 80.5% of the participants were diagnosed with unstable angina and 19.5% with acute MI. The group with combined Type D personality and depression showed the lowest percentage of perceived good health status (*p* = 0.010).

### 3.2. Patterns of Medication Adherence at 1- and 3-Month Follow-Ups

We classified medication non-adherence as a score of <6; the frequency by baseline Type D personality and depression at 1- and 3-month follow-ups is demonstrated in Table 2. The total prevalence of medication non-adherence at the 1- and 3-month follow-ups was 14% and 16% respectively. The baseline Type D personality and depression and depression-only groups displayed significantly high percentages (23.4% and 24%, respectively) of medication non-adherence at 1 month (*p* = 0.049). The Type D personality-only group displayed the highest (39.1%) medication non-adherence at the 3-month follow-up (*p* = 0.013).

We used repeated measures ANOVA for mean comparisons of medication adherence among the 4 groups, presented in Table 3. Medication adherence revealed statistically significant interactions by times and groups (*p* = 0.004). Medication adherence demonstrated statistically significant differences among the 4 groups (*p* = 0.025). Especially, at a mean of 6.49, medication adherence was significantly lower in the depression group as compared to the non-Type D personality and non-depression group at 1 month (*F* = 3.638, *p* = 0.013). At 3 months, with a mean of 5.96, medication adherence was significantly lower in the Type D personality group compared to the non-Type D personality and non-depression group (*F* = 3.688, *p* = 0.013).

### 3.3. Association between Medication Non-Adherence at Follow-Up and Type D Personality and Depression at Baseline

The relationships between low medication adherence and combined Type D personality and depression are shown in Table 4. Odds ratios (ORs) were calculated by unadjusted and adjusted logistic regression with all significant variables—age, sex, education, exercise, and perceived health status—among the 4 groups.

At 1 month, the depression-only group’s medication non-adherence rate was 2.639 times higher (95% confidence interval (CI) 1.170–5.953) than that of the reference group, which was composed of individuals with non-Type D personality and no depression. After adjustment, the medication non-adherence rate was 2.605 times higher (95% CI 1.083–6.266) than that of the reference group.

At 3 months, the Type D personality group had a low medication adherence rate that was 4.564 times higher (95% CI 1.749–11.913] than that of the reference group. After adjustment, the low medication adherence rate was 5.089 times higher (95% CI 1.881–13.771) than that of the reference group.

## 4. Discussion

Medication adherence after PCI is a crucial factor to prevent major adverse outcomes such as rehospitalization and mortality [2,3,4]. Recently, Type D personality and depression have been reported as independent predictors to improve health outcomes including medication adherence, quality of life, and survival rates in patients with cardiovascular disease [4,15,38]. The primary aim of this study was to investigate how combined Type D personality and depression affects medication adherence in patients after successful PCI. Our main findings showed that combined, these variables had a negative influence on medication adherence in patients after PCI. Also, we found higher rates of medication non-adherence in alone or combined Type D personality and depression groups than in the normal group.

With respect to rates of medication adherence, the MMAS-8 cut-off to identify medication non-adherence was 6 [28,29,30]. In this study, the total prevalence of medication non-adherence was around 14% at 1 month and 16% at 3 months. Compared to previous studies [27,39], the total prevalence of medication non-adherence was higher in our study. In acute MI patients treated with PCI at 6 weeks, early medication non-adherence, reflected by a score of <6, was 4% [27] and in an intervention study using administration timing simplification protocol in cardiovascular disease patients in Korea, MMAS scores of <6 were 6–9% at baseline [39]. Internationally, however, medication non-adherence rates determined by using the MMAS-8 varied from 14.2–54% [40,41,42]. The reasons for this difference might be variations in healthcare utilization or medical services in each country, and perhaps even the differences in the background characteristics of the subjects [39,40]. Even though medication adherence should be consistent, it rapidly decreased after discharge [7]. Previously, many cross-sectional studies have been conducted to determine the various factors affecting medication adherence [29,39,40]. When we compared with rates of medication non-adherence by combined Type D personality and depression, there were different results due to different measurement periods. Patients with a combination of Type D personality and depression and those with only depression after PCI showed a higher prevalence of medication non-adherence at 1 month (23–24%), and those with Type D personality showed a higher prevalence (39.1%) at 3 months compared to other patients. Our findings on medication non-adherence in patients with Type D personality are consistent with those of similar studies. In these studies, subjects with Type D personality, defined as the trait-like combination of high levels of NA and SI, were more likely to have poor medication adherence than those with non-Type D personality [15,43]. In addition, depression is an important risk factor for impaired health status in chronic CAD [44] and is associated with medication non-adherence in patients with heart disease.

Most importantly, in the present study, medication non-adherence at 1 month was significantly affected only by depression and at 3 months only by Type D personality. After adjusted variables consistent with those of previous studies, such as males, low educational levels, limited exercise, and low perceived good health status, we measured the OR of medication non-adherence. For 1 month after discharge following PCI in this study, patients with depression were 2.6 times more likely to fail to take the medication than the normal group, and at 3 months, 5.1 times higher than the Type D personality group. These results were consistent with previous medication adherence studies about Type D personality and depression. In short, Type D personality alone was a significant predictor of poor medication adherence at 3 or 6 months after discharge [15,43]. Medication non-adherence in depressed patients was approximately 1.8–2.3 times higher than in non-depressed patients [38,40].

According to previous studies, many patients might develop depression during or after the diagnosis and treatment of diseases of the heart as a vital organ associated with life [45]. Those with depression could return to their normal mood state in 3 months of non-pharmacological treatment or usual care [45]. In a previous study, from Type D personality was a particularly significant factor in individual differences in risk, and it did not change with time [33]. On the contrary, another previous study reported that Type D personality before and after cardiac surgery could change in nearly 60% of patients [46]. The reasons for the varying relationship between Type D personality and medication adherence from month to month need to be identified in the future.

This present study is meaningful in that it is the first cohort study to examine the combined impact of Type D personality and depression on medication non-adherence during a 3-month follow-up period among PCI patients. However, there was no statistical significance in the relationship between medication non-adherence and combined Type D personality and depression in our study. According to previous studies on cardiovascular disease, Type D personality is highly associated with depression [47] and Type D personality and depression are associated with lower health-related quality of life [19]. Therefore, patients who have both Type D personality and depression could require more complex treatments [20,48].

In the present study, we chose a minimum of one- and three-month follow-up periods to identify medication adherence for two reasons: first, a one-month follow-up for medication adherence is a meaningful point in preventing short-term readmissions (e.g., 30-day readmission) after PCI treatment [49]; second, three months is the shortest period to meaningfully measure medication adherence, especially now that prescriptions with 90-day supplies are common. Based on the previous study, the percentage of the medication adherence declined rapidly following treatment initiation, with less than 45% of the medication adherence at three months [7]. In addition, patients had significantly better rates of adherence to medication at six weeks if they had first follow-up appointments made prior to discharge [27,50].

This study had some limitations. First, it was based on a small convenience sample, with small numbers of patients with CAD in each group. In future studies, it is necessary to increase the number of samples in conjunction with numerous hospitals in order to obtain many subjects with CAD. Second, the fact that measurements of depression, Type D personality, and medication adherence were self-reported is a big limitation because the findings might underestimate or overestimate the actual rates. Third, we measured Type D personality and depression at baseline with 4 groups. As the prevalence of patients with Type D personality or depression could change at various time points after discharge, regular screening for Type D personality and depression is needed to identify their association with medication non-adherence. Fourth, in this study Cronbach’s *α* of MMAS-8 was 0.58 and indicated little higher than a minimally accepted level as low as 0.50. As known, the MMAS-8 had reported various value of Cronbach’s *α* from 0.54 to 0.83 in previous studies [30,51,52,53,54,55,56]. This problem might be related with patients’ characteristics in this study. Namely, our participants were relatively older and have a lower level of education. As a result, self-reported MMAS-8 needs to be carefully explained and measured with elderly patients in future studies. Furthermore, subjective measures including self-reporting, healthcare professional assessments and objective measures such as pill counts and biochemical tests will have advantages and should be used in combination [57].

## 5. Conclusions

Our main findings showed that individual Type D personality and depression were risk factors for medication non-adherence in patients who underwent PCI. Therefore, screening for Type D personality and depression at baseline may be useful strategies to improve medication adherence in patients who undergo PCI. Furthermore, nurses should consider integrative person-centered approaches for patients with CAD with a focus on identifying meaningful subgroups by Type D personality and depression.

## Figures and Tables

**Table 1:** Baseline characteristics by Type D personality and depression groups.

Characteristics	*N* (%)					*p*
	Total (*n* = 257)	Non-Type D Personality and Non-Depression (*n* = 162)	Type D Personality Only (*n* = 23)	Depression Only (*n*= 47)	Type D Personality and Depression (*n* = 25)	
Sociodemographic variables						
Age, mean (SD)	62.32 (10.49)	62.09 (10.90)	59.43 (10.46)	64.04 (10.19)	63.20 (7.98)	0.355
Sex * male	173 (67.3)	114 (70.4)	19 (82.6)	25 (53.2)	15 (60.0)	0.044
female	84 (32.7)	48 (29.6)	4 (17.4)	22 (46.8)	10 (40.0)	
Education level * low	160 (62.3)	89 (54.9)	14 (60.9)	33 (70.2)	24 (96.0)	0.001
high	97 (37.7)	73 (45.1)	9 (39.1)	14 (46.8)	1 (4.0)	
Spouse, yes	206 (80.2)	136 (84.0)	18 (78.3)	33 (70.2)	19 (76.0)	0.194
Monthly income < 1,000,000 (KR ₩)	126 (49.0)	81 (50.0)	7 (30.4)	22 (46.8)	16 (64.0)	0.134
Job, no	121 (47.1)	77 (47.5)	6 (26.1)	24 (51.1)	14 (56.0)	0.159
Health insurance *, yes	254 (98.8)	161 (99.4)	23 (100.0)	46 (97.9)	24 (96.0)	0.415
Exercise, yes	136 (52.9)	95 (58.6)	13 (56.5)	22 (46.8)	6 (24.0)	0.010
Current smoking, yes	58 (22.6)	36 (22.2)	6 (26.1)	10 (21.3)	6 (24.0)	0.969
Alcohol drinking, yes	76 (29.6)	53 (32.7)	7 (30.4)	11 (23.4)	5 (20.0)	0.434
Clinical variables						
Diagnosis unstable angina	207 (80.5)	133 (81.2)	18 (78.3)	37 (78.7)	19 (76.0)	0.860
AMI	50 (19.5)	29 (17.9)	5 (21.7)	10 (21.3)	6 (24.0)	
Hypertension, yes	133 (51.8)	87 (53.7)	9 (39.1)	22 (46.8)	15 (60.0)	0.414
Diabetes, yes	88 (34.2)	52 (32.1)	6 (26.1)	23 (48.9)	7 (28.0)	0.114
Hyperlipidemia, yes *	14 (5.4)	6 (3.7)	2 (8.7)	4 (8.5)	2 (8.0)	0.453
Perceived health status, good *	222 (86.4)	147 (90.7)	21 (91.3)	36 (76.6)	18 (72.0)	0.010

* Fisher’s exact test; AMI, acute myocardial infarction; PCI, percutaneous coronary intervention; M, month.

**Table 2:** Frequency of medication non-adherence by Type D personality and depression groups at baseline.

Medication Non-Adherence, Yes (a Score of <6)	*N* (%)					*p*
	Total (*n* = 257)	Non-Type D and Non-Depression (*n* = 162)	Type D Personality Only (*n* = 23)	Depression Only (*n* = 47)	Type D and Depression (*n* = 25)	
One month follow-up *	36 (14.0)	17 (10.5)	2 (8.7)	11 (23.4)	6 (24.0)	0.049
Three month follow-up *	41 (16.0)	20 (12.3)	9 (39.1)	8 (17.0)	4 (16.0)	0.013

* Fisher’s exact test; The MMAS (8-item) content, name, and trademarks are protected by US copyright and trademark laws. Permission for use of the scale and its coding is required. A license agreement is available from Donald E. Morisky, ScD, ScM, MSPH, 14725 NE 20th St Bellevue, WA 98007, USA; dmorisky@gmail.com.

**Table 3:** Mean score for medication adherence by Type D personality and depression at baseline.

Variables	Mean (SD)			*F*	*p*
	1 Month	3 Months			
Non-Type D personality and non-depression	7.09 (1.14)	6.95 (1.34)	Time	0.559	0.455
Type D personality only	6.96 (0.98)	5.96 (1.72) *	Group	3.160	0.025
Depression only	6.49 (1.52) *	6.83 (1.34)	Time * Group	4.501	0.004
Type D personality and depression	6.56 (1.36)	6.96 (1.06)			
*F*	3.638	3.688			
*p*	0.013	0.013			

Compared with normal group * *p* < 0.05.

**Table 4:** Odds ratios (OR) for the association with medication non-adherence by Type D personality and depression at baseline.

Variables	Unadjusted		Adjusted	
	OR (95% CI)		OR (95% CI)	
	1 Month	3 Months	1 Month	3 Months
Non-Type D personality and non-depression	1	1	1	1
Type D personality only	0.812 (0.175–3.770)	4.564 (1.749–11.913) *	0.773 (0.164–3.640)	5.089 (1.881–13.771) *
Depression only	2.606 (1.123–6.047) *	1.456 (0.596–3.558)	2.605 (1.083–6.266) *	1.472 (0.584–3.713)
Type D personality and depression	2.693 (0.946–7.669)	1.352 (0.421–4.345)	2.294 (0.734–7.167)	1.614 (0.461–5.654)

* *p* < 0.05; OR, odds ratio; CI, confidence interval; Adjusted for age, sex (male = 1, female = 0), education (low = 1, high = 0), exercise (yes = 0, no = 1), perceived health status (low = 1, high = 0).
